# Health Sciences Students’ Perceptions of the Role of the Supervisor in Clinical Placements

**DOI:** 10.3390/ijerph18094427

**Published:** 2021-04-21

**Authors:** Álvaro Borrallo-Riego, Eleonora Magni, Juan Antonio Jiménez-Álvarez, Vicente Fernández-Rodríguez, María Dolores Guerra-Martín

**Affiliations:** 1 Department of Nursing, Faculty of Nursing, Physiotherapy and Podiatry, University of Seville, 41009 Seville, Spain; alvaroborrallo@hotmail.com; 2 Department of Physiotherapy, Faculty of Nursing, Physiotherapy and Podiatry, University of Seville, 41009 Seville, Spain; magni@us.es; 3 Osuna University School, Teaching Center Attached to the University of Seville, 41640 Seville, Spain; jajimenez@euosuna.org; 4 Andalusian Health Service, Seville Health District, Mallén Health Center, 41018 Seville, Spain; vicente.fernandez.r.sspa@juntadeandalucia.es

**Keywords:** health sciences, health occupations students, perception, clinical clerkship, preceptorship

## Abstract

The supervision of clinical placements is essential to achieving a positive learning experience in the clinical setting and which supports the professional training of those being supervised. The aim of this study was to explore health sciences students’ perceptions of the role of the supervisor in the supervision of clinical placements. A quantitative methodology was used, administering a previously validated questionnaire, by means of an expert panel and a pre-test, to 134 students from the Faculty of Nursing, Physiotherapy and Podiatry at the University of Seville (Spain). The analysis of variables was carried out by means of a data matrix. The results revealed a statistically significant difference in the perception of placement supervision depending on the degree, with Nursing producing the highest degree of affirmation in the variables studied and the greatest satisfaction with placement supervision; in contrast, Physiotherapy produced the greatest dissatisfaction and the lowest degree of affirmation. The study and analysis of these perceptions facilitates the collection of relevant information in order to formulate actions that help to improve the supervision experience during placements. They also allow a greater understanding of what factors most influence the experience of supervision during clinical placements.

## 1. Introduction

During university training in health sciences, clinical placements are one of the main components for acquiring knowledge, skills and competences for the personal and professional development of students [1,2]. To enrich these experiences, the role of clinical supervisors, experienced professionals who provide support and promote the learning and professional development of their students, is indispensable [3,4,5]. In this way, university knowledge is integrated with professional experience. In some modalities, a peer or colleague of the student from the same course or a more advanced course and who has more experience and knowledge may take on the role of supervisor [3,6].

The role of the clinical supervisor has adapted with the times, changing from strictly in-person supervisors to the inclusion of virtual supervisors. This has led to a change in terms of accessibility, communication and interaction between the supervisor and the student during clinical placements [7,8].

Health sciences students report that in many cases the role of the clinical supervisor is not clearly defined, and they are unaware of their functions, the training needs of their students and the influence that this has on the learning process in clinical placements [4,9]. In this context, different authors have stated the need to create a clinical supervision system where the duties, available resources, existing limitations and the objectives to be achieved are clearly defined and identified, with a joint dialogue between students and clinical supervisors being indispensable [6,10,11].

Both clinical supervisors and students of health sciences agree that there are several factors that affect the supervision process of clinical placements, from the student’s motivation to learn and the supervisor’s interest in teaching, to others such as interpersonal relationships, communication skills, the learning environment and excessive workload. Each of these factors will largely affect the quality perceived by students in terms of learning and supervision in clinical placements [7,12,13]. We must not forget that the supervision of clinical placements is a collective responsibility between students and supervisors, with active involvement by both parties where the clinical supervisor is not limited to the mere transmission of knowledge and professional experience but guides their students in the construction of this knowledge, encouraging critical thinking, autonomy and self-learning [3,14]. The aforementioned point helps us to understand the complex reality in which clinical placements take place and highlights the need to understand and analyse the experience of health sciences students in terms of the supervision of clinical placements [12,15,16]. This is the reason why this study was carried out, with the main objective being to explore Nursing, Physiotherapy and Podiatry students’ perceptions of the role of the supervisor in clinical placements and to examine how this experience is interpreted depending on the academic degree.

Some authors have shown interest in analysing Health sciences students’ perception regarding the clinical supervisor role and practice environment. On the one hand, Vizcaya, Pérez, Jiménez and De Juan [9] made a study of which the main objective was to identify Nursing students’ perceptions of the clinical supervision and work environment during clinical practices. They exposed that the learning offered by the clinical supervisor was not individualised and the students’ experience widely depends on the teamwork and the supervisor assigned, whose role is usually not clearly defined. On the other hand, Guerra-Martín, Lima-Rodríguez and Lima-Serrano [17] made a study of which the aim was to analyse the Nursing students’ perceptions regarding their satisfaction with tutoring process. It showed that the satisfaction is related to the benefit obtained by the students from these meetings and its potential to, among other values, meet academic and professional needs.

## 2. Materials and Methods

### 2.1. Design and Participants

A quantitative, non-experimental and cross-sectional study was carried out. The study population consisted of 205 students enrolled in the final year of the Nursing, Physiotherapy and Podiatry degrees at the University of Seville during the 2018–2019 academic year.

The common point between the three selected degrees is the location, being part of the Faculty of Nursing, Physiotherapy and Podiatry. The Nursing and Physiotherapy degrees develop their clinical placement among the public health institutions while Podiatry can benefit from its own podiatric clinic inside the faculty. In these three degrees the role of the supervisor is to oversee the learning process during the clinical placement of the students.

**Figure 1:**
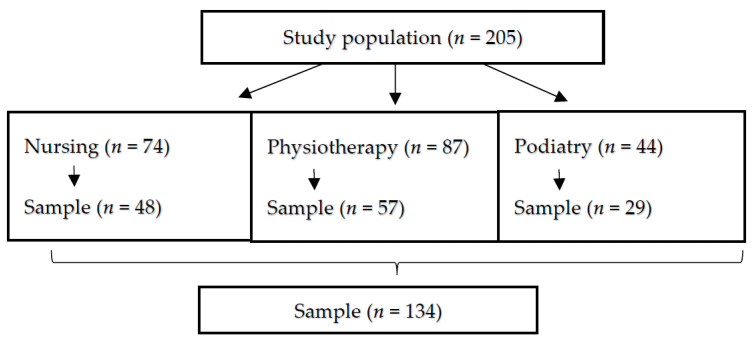
Study participants.

The sampling technique used was stratified probability sampling in accordance with the degrees for which the students were studying [18]. The sample, calculated at *p* < 0.05, consisted of 134 students (Figure 1).

### 2.2. Study Procedure

**Figure 2:**
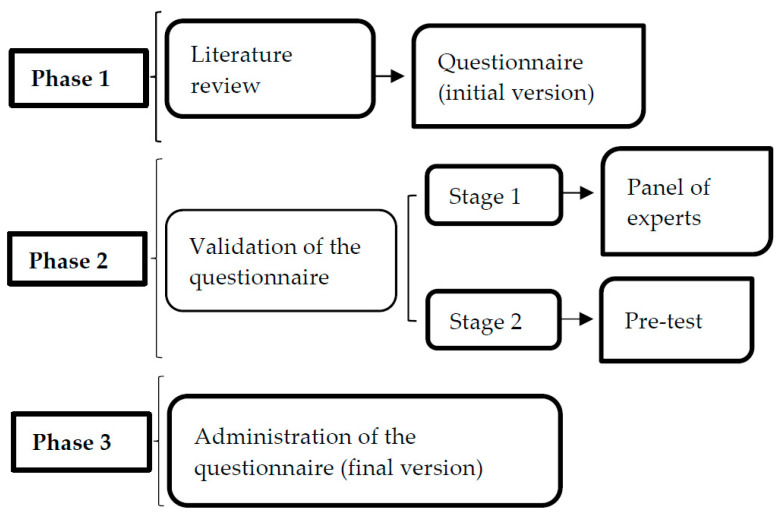
Phases of the study.

Figure 2 shows the phases of the study.

Phase 1: A literature review about this research topic was developed using the databases PubMed, CINAHL, PsycINFO y WOS. The following inclusion criteria were adopted: Quantitative and qualitative research studies, published in Spanish or English, from 2013 to 2018. The same searching strategy [18] was used in the different databases: (Perception AND “Health Occupations Students” AND (Preceptorship OR “Clinical Clerkship”)). A total of 85 studies were found. By means of a first screening, titles and abstracts were read in pairs, selecting the concordant studies. During the second screening, the full-texts of previously selected studies were read, selecting 11 of them [2,12,15,16,19,20,21,22,23,24,25].

After reviewing the scientific literature on questionnaires that analysed the supervision of clinical practices, the questionnaire by Palacios and Quiroga [19] on the analysis of clinical teaching in the Faculty of Dentistry of the University of Concepción (Chile) according to the perception of the students, was selected because it identifies and prioritises the teaching conditions and behaviours of the supervisor related to learning in the clinical setting. The questionnaire is grouped into different dimensions of teaching, each of which has different questions associated with it (34 in total).

In the study of Palacios-Gutiérrez et al. [19], data collection was carried out through questionnaire administration. Furthermore, the data were transferred to a template in Microsoft Excel and analysed using the IBM SPSS Statistics 19 tool. For the results, two evaluation criteria were considered: level of approval and level of achievement. For the Approval Level, the percentage of students who rated the items of the questionnaire was considered. For the level of achievement, the weighted percentage of the grades obtained was considered. The R-Pearson test was used for correlation. Additionally, the comments of the students were taken into account.

Phase 2: The questionnaire was validated in two stages. In the first stage, an expert panel review process was conducted for content validity [18,26]. The following criteria were taken into account: relevance, coherence, structuring, clarity, applicability and universality, which were assessed using a five-point Likert-type scale (1. Never. 2. Almost never. 3: Sometimes. 4. Almost Always. 5. Always), including an open-ended question at the end for relevant comments and/or suggestions for improvement [3]. For an item to be considered as valid, the criterion had to have a value between 4 and 5 and a percentage equal to or greater than 80%, and if this guideline was not met, the standard deviation was assessed to be equal to or less than 0.90 [3,27]. The next stage began when the criteria were met. There were 12 experts, four from each degree (Nursing, Physiotherapy and Podiatry) from the University of Seville (US), and 50% of them were women.

In the second stage, a pre-test was conducted with students to assess the suitability of the questionnaire [18]. There were three groups, one for each degree involved in the study, with six students from Nursing, four from Physiotherapy and four from Podiatry. 64.3% of the participants were women. The data were collected in the classroom with the teacher’s consent. Once the data obtained from the pre-test had been analysed, the necessary modifications, adjustments and improvements were made for the final configuration of the questionnaire (change to the order of the dimensions so that the questions had a common thread; including the option: “I don’t know” in the answers). Table 1 shows the dimensions and questions.

Furthermore, the students expressed their desire for their perceptions of the supervision of placements to be investigated, for which a closed-ended question on satisfaction and an open-ended question for comments and/or suggestions were included.

Phase 3: Subsequently, the final version of the questionnaire was administered to the study sample of 134 students. After this, we proceeded to collect, code, analyse and present the results.

### 2.3. Data Analysis

The variable analysis was carried out on the data matrix using the SPSS statistical package for Windows (v.19.0. IBM Corp., Armonk, NY, USA). For the descriptive analysis, the qualitative variables were characterised in different frequency distribution tables and percentages, and for the quantitative variables by means of measures of centralisation and dispersion. The Kruskal–Wallis test was used for the hypothesis testing, establishing those with *p* < 0.05 values as statistically significant differences. Cronbach’s alpha coefficient was calculated in order to determine the reliability of the instrument. For the open-ended question at the end of the questionnaire, a qualitative and categorical analysis of the textual data was carried out [18]. Comments were sorted into seven categories.

### 2.4. Ethical Considerations

The study was conducted in accordance with the Declaration of Helsinki. Prior to the start of the study, permission was requested from the authors of the original study to obtain their approval and consent to the use of the questionnaire. In the context of our work, it is not necessary to request permission from the ethics committee for educational research. This is only needed for clinical research. Therefore, permission was requested from the Dean of the Faculty of Nursing, Physiotherapy and Podiatry of the University of Seville where the study was carried out (Reference MF/CF-29 of March 2017, no. 298). Informed consent was also requested from the students. Participation was voluntary and anonymous, ensuring the confidentiality of the data.

## 3. Results

### 3.1. Sociodemographic Characteristics of the Participants

Of the total student population, 134 students with a mean age of 22.59 years (SD = 2.58), and who were predominantly female (75.37%), participated in the study. The data stratified according to degree are shown in Table 2.

### 3.2. Health Sciences Students’ Perceptions of the Role of the Supervisor in Clinical Placements

Table 3 shows the comparative analysis of the data obtained in each of the degrees of the study according to the dimensions previously mentioned in the methodology. All degree programmes agreed that the clinical supervisors carry out their activities in an organised way during the clinical placements, being punctual and available to pay attention to their students. However, in Physiotherapy, the students specified that the clinical supervisors do not usually go beyond their care duties to pay attention to the supervised students, with a great discrepancy with Nursing and Podiatry where this affirmation was lower. Similarly, in Nursing and Podiatry, more students than in Physiotherapy affirmed that the clinical supervisors offered guidance to the students, being accessible, communicative, clear in the presentation of objectives and open to different opinions.

To a greater degree than Physiotherapy and Podiatry students, Nursing students affirmed that the clinical supervisor acts as a learning model who is interested in teaching, exhibiting knowledge and clinical skills and communicating successes and mistakes to their students, thus creating a positive atmosphere during the supervision of the placement. The placement environment is clearly affected by the supervisor–student relationship and requires respect between the parties and partiality in treatment. Podiatry degree students stated that this was the case to a lesser extent. All degree programmes stated that the clinical supervisors allow them to act with autonomy and independence, protecting the safety of the students themselves and of the patients.

Statistically significant differences (*p* < 0.05) were detected in the data obtained according to the degree of origin. Nursing was the one that reflected a higher degree of affirmation in the generality of the variables of the study. On the other hand, physiotherapy students showed the smallest degree of affirmation in the majority of these same variables.

Table 4 shows the average range obtained in each degree according to the teaching dimension. For the average range, all ratings were added and divided by the total number of students in each degree, allowing us to obtain an idea of the trend in each group.

Nursing students responded with a higher average range in most of the items, providing a more positive assessment of the role of the supervisor in clinical placements. Furthermore, the Physiotherapy degree responded with a lower average range to most of the items, which indicates greater dissatisfaction with the role of the supervisor in clinical placements.

### 3.3. Instrument Reliability

The Cronbach’s alpha coefficient was 0.9558, which means an excellent reliability [18].

### 3.4. Degree of Student Satisfaction

Table 5 shows the results pursuant to the degree of satisfaction with the supervision of the clinical placement according to the degree. Around 65% of the Nursing students were quite or very satisfied with the supervision of placements; in Podiatry, this statistic was 45%, and in Physiotherapy slightly less than 20%, giving a statistically significant difference (*p* = 0.00).

### 3.5. Comments and/or Suggestions from Students

Of the total sample, 87 students (64.95%) answered the open-ended question about their experience with the placement supervision, with 33 of them (24.62%) offering more than one comment. In total, there were 120 comments. A total of 22 were positive and 98 were negative. Students from the Physiotherapy degree provided the most contributions. These comments were grouped into seven categories. Some examples of which are shown in Table 6.

In general, the role of the clinical supervisor as a learning model was described by the students with a certain degree of neglect and absence, with the students feeling alone in the clinical setting and carrying out substitute work. Many of the comments also reflected expressions of disinterest and/or lack of motivation displayed by the clinical supervisor when teaching, and in said comments the supervisor was portrayed to have poor communication skills, especially with regard to resolving doubts, clearly defining the evaluation criteria or indicating whether the procedures and/or techniques are being performed correctly or not. In this context, there were some comments that mentioned that in order to break down communication barriers it would be advisable to incorporate telephone or email contact with the clinical supervisor, especially in cases of unforeseen events and/or problems that may occur during the placement.

One of the aspects that stood out the most among the comments was the work environment. Many of the students described the importance of creating a positive placement environment that is free of tension, allowing them to work in a calm and comfortable environment. The clinical supervisor was identified as a key figure in facilitating an appropriate placement environment, with maintaining mutual commitment and respect being important.

The organisation of clinical placements was another issue that stood out in the comments. There were notable differences depending on the degree: In Nursing, they focused on organisational changes in terms of whether to carry out placements in hospital services or in primary care, the latter being more highly rated. In addition, they believe that the role of the clinical supervisor is not well defined in many services, causing inconveniences in their development and assessment; in Physiotherapy, the comments focused on the lack of control and organisation of placement periods, with some stating the need to increase placement periods in private institutions to the detriment of public ones, considering that the latter would be more in line with the reality of work after completing their studies; in Podiatry, they reflected the need to improve the distribution of placement periods and timetables.

## 4. Discussion

The sociodemographic characteristics of the study population are similar to those found in other studies [3,22,28], which had a high participation of women, almost 80%, and with a mean age around 22 years [3,28].

According to the results, the students highlighted that the correct organisation and planning of the placement is essential to enjoying the learning process in the clinical setting. These data corroborate the findings of other studies [10,11], which consider that both students and clinical supervisors should be jointly involved in the organisation of placements to ensure success and satisfaction. In this sense, the students defend the need to have a clinical supervisor who is accessible and available to the students during the placement period, showing knowledge and advice, especially when it comes to resolving doubts and/or difficulties. These data coincide with what has been stated by other authors [29,30,31] who establish the willingness and accessibility of the clinical supervisor as guarantors in the orientation and guidance of those being supervised.

Almost all of the participants in the study claimed that the clinical supervisor should show and teach their students current, evidence-based techniques and procedures that are relevant to their training and that help them to achieve their professional goals. This is consistent with the findings of other authors [32,33]. Similarly, clinical supervisors should be shown as learning role models who inspire students to complete their training [33,34,35], as witnessing inappropriate learning role models negatively affects the experience of those being supervised in the clinical setting [30].

Regarding communication skills during the supervision of the placement, the results obtained are in line with other authors [31,36,37], who emphasise that fostering communication skills improves and helps the development of the supervisor–student relationship. In addition, among the improvements for tutorial action proposed by the students in the study, the need to clearly and precisely communicate what is expected of them, and what objectives and goals are expected to be met is described, coinciding with what has been described by other authors [31,38]. According to the results, the supervisor–student relationship should not only be based on communication, but also on mutual respect, which will foster trust and feedback between the parties, in line with the results of other studies [20,39].

The supervisor–student relationship is clearly influenced by the working environment during the placement, and a positive atmosphere is necessary for both parties to get the most out of the clinical experience. Many authors have shown that a safe, pleasant, calm and permissive environment allows the student to feel part of the team, enriching the learning experience [29,31]. This positive space should also allow the student to assume a certain degree of autonomy to take responsibility for their own learning, therefore fostering independence and critical thinking [7].

The results show that in order to fulfil many of the aspects outlined above, a certain willingness and interest in teaching on the part of the clinical supervisor is required. Several studies postulate that there must be a genuine willingness and mutual interest in learning, considering that the greater the interest in teaching, the better the learning outcomes [13,29,32].

Regarding satisfaction with the supervision of clinical placements, in Nursing and Podiatry it was higher than in other studies [3,17,40], while in Physiotherapy these were similar to the study by Pérez [41] where the responses indicating high or very high satisfaction were just over 18%.

According to students’ suggestions, some improvement strategies were proposed. The first one is related to the clinical supervisor, who should show motivation and interest in teaching; therefore, this is a value to consider on the supervisor–student assignment. The second one expresses the need to build on clinical supervisors’ communication skills in order to improve the communication and feedback with their students. The third one suggests promoting an appropriate tension-free work environment. These aspects are suitable to be considered for future studies on each analysed degree.

Furthermore, theoretical and practical implications can be found for this study. Regarding the theoretical implications, they are the need to highlight an agreement for clinical supervisors’ role, which is not always well defined. Therefore, it is necessary to find out more about this role, focusing on the functions a clinical supervisor must perform, which is in line with other authors’ suggestions [17]. Practical implications have been described and discussed along the current study, emphasising the importance of communication skills, the feedback with the student, to offer some autonomy level which encourages each student’s individual learning growth, to promote the collaborative learning, to make students participate in the clinical placement planning and to let them know beforehand what skills they should gain during clinical placements.

### Limitations

Among the limitations of the study are those related to the descriptive approach, together with the fact that only one measurement of the phenomenon was made, which may have caused a lack of control of factors that could invalidate the research. In addition, questionnaire data collection is not free of bias. Closed-ended questions limit the answers, which can lead to the exclusion of relevant information. In the open-ended questions, the limitation may be due to the categorisation of the comments made by the students. Finally, limitations related to the sample size must be taken into account.

## 5. Conclusions

Statistically significant differences were detected between the degrees when analysing the supervision of clinical placements. Nursing students rated the dimensions of the questionnaire more highly than those of Physiotherapy and Podiatry, as well as being more satisfied with the supervision of the placements. The Physiotherapy students valued the different dimensions the worst and were the ones who said they were least satisfied and who made the most comments when they were asked the open-ended question (mostly negative). If lecturers and academic managers know the students’ opinions about the role of the supervisor in clinical placements, this can contribute to the proposal of measures to improve the supervision of placements and student satisfaction.

## Figures and Tables

**Table 1:** Questionnaire on the role of the supervisor in placements.

Item	Dimensions	Questions Relating to the Clinical Supervisor on the Supervision of Placements
1	Organisation	Q1: Are they punctual and available at scheduled times? Q2: Do they carry out their activities in an organised way?
2	Environment	Q3: Do they create a positive, stress-free, tolerant, calm and patient learning environment?
3	Professionalism	Q4: Are they a role model for professional competence? Q5: Do they take their job as a teacher seriously?
4	Communication skills	Q6: Are they expressive, both verbally and non-verbally? Q7: Are they accessible and communicative?
5	Clarity and comprehensibility	Q8: Do they clearly communicate the behaviours, roles, and performance they expect from students? Q9: Do they explain concepts and techniques clearly and succinctly? Q10: Do they answer questions in detail?
6	Feedback with the student	Q11: Do they analyse students’ work by indicating what they do correctly and incorrectly? Q12: Do they provide frequent and constructive feedback? Q13: Do they explain to students why their work is not acceptable?
7	Student autonomy	Q14: Do they allow students to act as independently as possible, preserving the safety of students and patients?
8	Respect for students	Q15: Are they respectful, do they not make students feel intimidated or out of place? Q16: Do they question the student’s judgement and/or abilities in front of patients? Q17: Do they praise students’ work in front of patients, teachers and/or peers?
9	Perceived achievement in teaching	Q18: Do they teach current and relevant clinical concepts and procedures?
10	Teacher equity	Q19: Are they impartial in their treatment, do they treat students without favouritism? Q20: Do their grades reflect the quality of student work?
11	Enthusiasm for the subject and for teaching	Q21: Do they enjoy teaching, showing interest in student learning?
12	Availability and willingness to help	Q22: Are they hardworking and do they go above and beyond their care obligations? Q23: Do they guide students, providing advice and suggestions? Q24: Are they available and responsive?
13	Knowledge of the subject matter	Q25: Do they exhibit clinical knowledge and skills commensurate with their area of speciality? Q26: Do they demonstrate or perform procedures on patients as a model?
14	Intellectual challenge	Q27: Do they motivate the student to perform to the best of their ability?
15	Stimulation of interest in the course and its content	Q28: Do they make learning interesting, enjoyable and fun?
16	Encouraging discussion and opinion	Q29: Are they flexible and open to different points of view? Q30: Is the clinical supervisor able to critique their own performance? Q31: Do they actively listen to students?
17	Awareness of the level of the class and its progress	Q32: Do they consider the student’s level of knowledge and experience when teaching? Q33: Do they show interest in difficulties students may have? Q34: Do they motivate students and ask how the work is going?

Source: based on Palacios and Quiroga [19].

**Table 2:** Distribution of students according to degree, average age and female.

Degree	Total (%)	Average Age (SD)	Female (%)
Nursing	48 (35.82)	22.60 (3.24)	43 (89.58)
Physiotherapy	57 (42.54)	22.68 (2.42)	38 (66.66)
Podiatry	29 (21.64)	22.31 (1.49)	20 (68.98)

SD: Standard deviation.

**Table 3:** Results obtained in each degree according to the teaching dimension. *p*-value. Kruskal–Wallis test.

TD	Degree. Frequency (%)									*p*	K
	Nursing			Physiotherapy			Podiatry				
	N/AN	S	A/AA	N/AN	S	A/AA	N/AN	S	A/AA		
1. Q1	3 (6.2)	5 (10.4)	40 (53.4)	0 (0)	14 (24.6)	43 (75.4)	0 (0)	6 (20.7)	23 (79.3)	0.18	3.3
Q2	4 (8.3)	14 (29.1)	30 (62.5)	1 (1.7)	25 (43.9)	31 (54.4)	4 (13.8)	10 (34.4)	15 (51.7)	0.18	3.4
2. Q3	3 (6.3)	12 (25)	31 (64.6)	9 (15.8)	32 (56.1)	16 (28.1)	2 (6.9)	19 (65.5)	8 (27.6)	0	15.4
3. Q4	3 (6.3)	17 (35.4)	27 (56.3)	25 (43.9)	28 (49.1)	3 (5.3)	2 (6.9)	15 (51.7)	12 (41.4)	0	40.9
Q5	8 (16.6)	12 (25)	28 (58.4)	17 (29.8)	31 (54.4)	9 (15.8)	0 (0)	14 (48.3)	15 (51.7)	0	24.8
4. Q6	0 (0)	10 (20.8)	38 (79.2)	4 (7)	23 (40.4)	30 (52.6)	0 (0)	11 (38)	18 (62)	0	9.2
Q7	3 (6.2)	13 (27.1)	32 (66.7)	19 (33.3)	30 (52.6)	8 (14.1)	3 (10.3)	16 (55.1)	10 (34.15)	0	34.1
5. Q8	8 (16.6)	19 (39.6)	21 (43.7)	30 (52.6)	20 (35.1)	7 (12.2)	2 (6.9)	12 (41.4)	15 (51.7)	0	31.9
Q9	4 (8.3)	14 (29.1)	29 (60.4)	10 (17.5)	32 (56.1)	15 (26.3)	1 (3.5)	16 (55.1)	12 (41.4)	0	15.2
Q10	4 (8.3)	17 (35.4)	27 (56.3)	6 (10.5)	30 (52.6)	21 (36.9)	1 (3.5)	15 (51.7)	13 (44.8)	0.07	5.2
6. Q11	3 (6.3)	14 (29.1)	31 (64.6)	9 (15.8)	33 (57.9)	14 (24.6)	13 (44.8)	6 (20.7)	10 (34.4)	0	19.3
Q12	5 (10.4)	12 (25)	31 (64.6)	19 (33.3)	28 (49.1)	10 (17.5)	7 (24.1)	14 (40.3)	8 (27.6)	0	26.7
Q13	5 (10.4)	25 (12.1)	17 (35.4)	22 (38.6)	24 (42.1)	10 (17.5)	12 (41.4)	8 (27.6	9 (31.1)	0	10.7
7. Q14	4 (8.3)	9 (18.7)	34 (70.8)	5 (8.8)	17 (29.8)	35 (61.4)	3 (10.3)	8 (27.6)	18 (62)	0.26	2.7
8. Q15	1 (2.1)	8 (16.6)	39 (81.3)	4 (7)	14 (24.6)	39 (68.4)	3 (10.3)	7 (24.1)	19 (65.5)	0	10.8
Q16	23 (47.9)	17 (35.4)	5 (10.4)	32 (56.1)	20 (35.1)	5 (8.8)	10 (34.4)	14 (48.3)	5 (17.21)	0.17	3.5
Q17	6 (12.5)	16 (33.3)	26 (54.1)	23 (40.3)	27 (47.4)	6 (10.5)	8 (27.6)	14 (48.3)	6 (20.7)	0	25.6
9. Q18	7 (14.6)	11 (23)	29 (60.4)	22 (38.6)	22 (38.6)	13 (22.8)	4 (13.8)	17 (58.6)	8 (27.6)	0	18.8
10. Q19	4 (8.3)	10 (20.9)	32 (68.8)	9 (15.8)	17 (29.8)	31 (54.4)	6 (20.7)	12 (41.4)	11 (38)	0	9.5
Q20	4 (8.3)	11 (23)	30 (62.5)	22 (38.6)	22 (38.6)	13 (22.8)	8 (27.6)	15 (51.7)	6 (20.7)	0	20.7
11. Q21	6 (12.5)	16 (33.3)	25 (52.1)	23 (40.4)	29 (50.9)	4 (7)	3 (10.3)	20 (69)	6 (20.7)	0	26.4
12. Q22	6 (12.5)	18 (17.5)	24 (50)	24 (42.1)	22 (38.6)	11 (19.3)	3 (10.3)	14 (48.3)	12 (41.4)	0	19.3
Q23	6 (12.5)	17 (35.4)	25 (52.8)	13 (22.8)	28 (49.1)	16 (28.1)	5 (17.2)	10 (34.4)	14 (48.3)	0.02	7.4
Q24	8 (16.6)	16 (33.3)	24 (50)	15 (26.3)	23 (40.3)	18 (33.4)	1 (3.5)	15 (51.7)	12 (41.4)	0.06	5.3
13. Q25	2 (4.2)	8 (16.6)	38 (79.2)	12 (21.1)	27 (47.4)	18 (31.5)	2 (6.9)	8 (27.6)	19 (65.5)	0	31.9
Q26	2 (4.29	10 (20.9)	33 (68.8)	11 (19.3)	22 (38.6)	23 (40.3)	7 (24.5)	11 (38)	10 (34.4)	0	12.8
14. Q27	4 (8.3)	16 (33.3)	27 (56.3)	18 (33.4)	31(54.4)	7 (12.2)	7 (24.1)	12 (41.4)	10 (34.5)	0	22.3
15. Q28	3 (6.3)	21 (43.7)	24 (50)	23 (40.4)	31 (54.4)	3 (5.3)	3 (10.3)	17 (58.6)	9 (31.1)	0	36.2
16. Q29	4 (8.3)	17 (35.4)	26 (54.1)	6 (10.5)	22 (38.6)	29 (50.9)	4 (13.8)	12 (41.4)	13 (44.8)	0.55	1.2
Q30	8(16.7)	19 (39.6)	20 (41.7)	30 (52.7)	22 (38.6)	4 (7.01)	8 (27.6)	11 (37.9)	9 (31.1)	0	22.2
Q31	5 (10.4)	12 (25)	31 (64.6)	30 (52.7)	22 (38.6)	4 (7.01)	8 (27.6)	11 (37.9)	9 (31.1)	0	10.9
17. Q32	8 (16.6)	14 (29.1)	25 (52.1)	7 (12.2)	26 (45.6)	23 (40.4)	2 (6.9)	18 (62)	9 (31.1)	0.28	2.5
Q33	4 (8.3)	17 (35.4)	26 (54.1)	10 (17.5)	33 (57.9)	14 (24.6)	3 (10.3)	15 (51.7)	11 (38)	0	10.4
Q34	10 (20.9)	12 (259	26 (54.1)	26 (45.6)	22 (38.6)	7 (12.2)	10 (34.4)	11 (38)	8 (27.69	0	20.9

TD: Teaching dimension; N/AN: Never or almost never; S: Sometimes; A/AA: Always or almost always; *p*: *p*-value; K: Kruskal–Wallis test.

**Table 4:** Average range obtained in each degree according to the teaching dimension.

Teaching	Degree		
Dimension	Nursing (F: 48)	Physiotherapy (F: 57)	Podiatry (F: 29)
	Average Range (%)		
1. Q1	73.47	61.13	70.12
Q2	75.14	63.79	62.12
2. Q3	83.71	56.57	62.12
3. Q4	88.16	44.37	78.74
Q5	80.18	49.1	82.65
4. Q6	78.79	57.83	67.81
Q7	90.09	48.1	68.22
5. Q8	81.07	46.49	86.32
Q9	82.08	54.43	69.03
Q10	76.18	60.15	67.55
6. Q11	86.05	58.46	54.55
Q12	88.75	52.38	62.03
Q13	80.9	57.38	65.18
7. Q14	74.33	63.28	64.48
8. Q15	81.21	60.79	57.96
Q16	63.26	65.3	78.82
Q17	88.23	51.99	63.65
9. Q18	84.92	53.3	66.55
10. Q19	80.34	62.18	56.68
Q20	87.07	55.19	59.29
11. Q21	85.85	49.8	71.89
12. Q22	81.32	51.33	76.39
Q23	76.9	57.77	71.05
Q24	75.82	59.37	69.68
13. Q25	87.65	47.44	73.55
Q26	82.32	61.21	55.31
14. Q27	86.13	52.15	66.81
15. Q28	87.96	46.7	74.5
16. Q29	71.62	66.59	62.44
Q30	84.59	50.69	72.24
Q31	79.98	56.22	68.98
17. Q32	74.08	64.54	62.41
Q33	79.88	57.11	67.41
Q34	85.68	52.28	67.29

**F:** Frequency.

**Table 5:** Results of the degree of satisfaction with the supervision of placements depending on the degree.

Categories	Degree Frequency (%)		
	Nursing	Physiotherapy	Podiatry
Very dissatisfied	0 (0%)	1 (1.7%)	0 (0%)
Not very satisfied	1 (2.1%)	16 (28.1%)	3 (10.3%)
Somewhat satisfied	10 (20.8%)	29 (50.9%)	13 (44.8%)
Quite satisfied	31 (64.6%)	11 (19.3%)	12 (41.4%)
Very satisfied	6 (12.5%)	0 (0%)	1 (3.5%)

**Table 6:** Categories obtained after grouping the comments made by the students.

Category	Comments Made by Students
Role of clinical supervisor as a learning model	“[...] clinical supervisors do not act like a teacher, it’s as though we’re assistants to their work [...]” S.11 “[...] supervisors should review the work, telling us what we have done right and wrong [...]” S.117
Interest in teaching	“Some teachers have no interest in their students” S. 32 “There are good supervisors who care about teaching and about making sure we learn [...]” S.47
Communication between supervisors and students	“Most of them [...] are defensive and do not accept opinions on the work performed” S. 9 “I have not come across any placement teacher who has treated me badly [...]” S.104
Respect for students	“[...] Supervisors who look down on you and do not welcome you [...]” S.23 “...], I needed more availability in terms of clarifying doubts [...]” S.100
Placement assessment	“Grades and assessment criteria are not clear [...]” S.14 “[...] Grades almost never represent the student’s real effort” S.80
Work environment	“...] supervisors create a tense atmosphere that makes it difficult to work at ease [...]” S.110 “[...] supervisors encourage student learning by creating a positive environment [...]” S.129
Practical organisation	Nursing: “Primary care placements are better organised than hospital placements [...]” S.60 “There are services where there is no fixed supervisor, since you are with a different one every day [...]” S.96
	Physiotherapy: “there is a need for better organisation and control of clinical placements” S. 5 “More private centres would enrich learning and knowledge of the world of work [...]” S.8 Podiatry:
	“...] to distribute the placements better because we are often overburdened” S.110 “[...] bad schedules. Morning-afternoon placements are not benefited from because of fatigue [...]” S.131

## Data Availability

The data presented in this study are available on request from the corresponding author.
